# Deep learning automates detection of wall motion abnormalities *via* measurement of longitudinal strain from ECG-gated CT images

**DOI:** 10.3389/fcvm.2022.1009445

**Published:** 2022-12-15

**Authors:** Hui Li, Zhennong Chen, Andrew M. Kahn, Seth Kligerman, Hari K. Narayan, Francisco J. Contijoch

**Affiliations:** ^1^Department of Bioengineering, University of California, San Diego, La Jolla, CA, United States; ^2^Department of Medicine, Division of Cardiovascular Medicine, University of California, San Diego, La Jolla, CA, United States; ^3^Department of Radiology, University of California, San Diego, La Jolla, CA, United States; ^4^Department of Pediatrics, University of California, San Diego, La Jolla, CA, United States

**Keywords:** cardiac computed tomography, left ventricle, wall motion abnormality, longitudinal strain, image segmentation, deep learning

## Abstract

**Introduction:**

4D cardiac CT (cineCT) is increasingly used to evaluate cardiac dynamics. While echocardiography and CMR have demonstrated the utility of longitudinal strain (LS) measures, measuring LS from cineCT currently requires reformatting the 4D dataset into long-axis imaging planes and delineating the endocardial boundary across time. In this work, we demonstrate the ability of a recently published deep learning framework to automatically and accurately measure LS for detection of wall motion abnormalities (WMA).

**Methods:**

One hundred clinical cineCT studies were evaluated by three experienced cardiac CT readers to identify whether each AHA segment had a WMA. Fifty cases were used for method development and an independent group of 50 were used for testing. A previously developed convolutional neural network was used to automatically segment the LV bloodpool and to define the 2, 3, and 4 CH long-axis imaging planes. LS was measured as the perimeter of the bloodpool for each long-axis plane. Two smoothing approaches were developed to avoid artifacts due to papillary muscle insertion and texture of the endocardial surface. The impact of the smoothing was evaluated by comparison of LS estimates to LV ejection fraction and the fractional area change of the corresponding view.

**Results:**

The automated, DL approach successfully analyzed 48/50 patients in the training cohort and 47/50 in the testing cohort. The optimal LS cutoff for identification of WMA was −21.8, −15.4, and −16.6% for the 2-, 3-, and 4-CH views in the training cohort. This led to correct labeling of 85, 85, and 83% of 2-, 3-, and 4-CH views, respectively, in the testing cohort. Per-study accuracy was 83% (84% sensitivity and 82% specificity). Smoothing significantly improved agreement between LS and fractional area change (*R*^2^: 2 CH = 0.38 vs. 0.89 vs. 0.92).

**Conclusion:**

Automated LV blood pool segmentation and long-axis plane delineation *via* deep learning enables automatic LS assessment. LS values accurately identify regional wall motion abnormalities and may be used to complement standard visual assessments.

## Introduction

Longitudinal strain (LS), measured using echocardiography (1) or cardiac magnetic resonance (2), has been proven useful in evaluating patients at risk of chemotherapy cardiotoxicity (3) and those with aortic stenosis (4, 5), cardiac amyloidosis (6) atrial fibrillation (7), and heart failure patients (8). In revascularized STEMI patients, CMR-based LS was superior and incremental to LVEF and scar size in the prediction of MACE (9).

LS can also be used as a quantitative metric to improve detection of wall motion abnormalities (WMA) (10, 11) and in the setting of infarction WMA have been shown to be independent predictors of adverse events (12, 13). Further, in patients without overt cardiovascular disease, presence of a WMA leads to a 2.4–3.4 higher risk of cardiovascular morbidity and mortality, independent of established risk factors (14).

Cardiac computed tomography (CT) is increasingly used to evaluate both coronary artery anatomy (15, 16) and cardiac function (17). Recent work has shown that ECG-gated CT can detect regional wall motion abnormalities (18–21) and that findings agree with echocardiography (22, 23) and CMR (18, 24). However, quantitative evaluation of cardiac function on 4D CT data can require significant computational processing such as 3D segmentation or measurement of wall thickening.

While several automated methods have been developed for the evaluation of cardiac chamber size and global function (25–28), automated estimation of LS from 4DCT is not currently available as it requires the combination of manual/semi-automated reformatting of the 4D dataset into long-axis imaging planes as well as delineation of the endocardial boundary across frames (29).

Recently, a deep learning framework has been shown to automatically and accurately identify the long-axis planes within a 4D CT dataset and, using the same architecture, segment the LA and LV blood pools (30). Specifically, long-axis views generated *via* the DL method were in close agreement with user-defined planes and >94% of views were diagnostically accurate. By segmenting both the LV and LA blood pools, this creates the opportunity to evaluate LS by measuring the LV endocardial perimeter (after removal of the mitral valve plane).

In this study, we evaluate the ability of this recently developed deep learning algorithm to be adapted to obtain automated LS estimates from each long-axis view. To test the clinical utility of our approach, we evaluated whether automatic LS can be used to detect WMA in a set of 100 clinical cases which were visually analyzed by three trained experts for the presence of WMA. We created two cohorts (*n* = 50 training and *n* = 50 testing cases). We used the training cohort to determine the optimal LS threshold for detecting a WMA and report accuracy in the independent testing cohort.

## Methods

### Study population

This study was approved by our system's institutional review board with waiver of informed consent. Five hundred and five ECG-gated contrast enhanced cardiac CT studies were acquired between April 2018 and December 2020 which had (1) full R-wave to R-wave (RR) coverage and (2) an imaging report including the explicit mention of cardiac function as normal or abnormal (either globally or regionally) (Table 1). All CT scans were performed on the same wide-detector CT scanner with 256 detector rows and 16 cm *z*-axis coverage (Revolution scanner, GE Healthcare, Chicago IL).

**Table 1:** Patient cohort information.

	**Entire dataset**	**Training cohort**	**Validation cohort**
Cohort size, *n*	100	50	50
Age, years	59 ± 14	59 ± 15	59 ± 13
Male, %	61	58	64
Median LVEF, %	62.4 (IQR: 41.7–69.3)	62.1 (IQR: 38.9–69.6)	63.8 (IQR: 45.1–68.5)
Abnormal segments	27% (432/1,600)	27% (219/800)	27% (213/800)
Normal studies, *n*	54	28	26
Study indication, *n*			
Coronary disease	50	21	29
Pulm. vein ablation	33	19	14
Heart failure	9	4	5
Aortic stenosis	5	4	1
Cardi-oncology	3	2	1

LVEF, left ventricular ejection fraction.

Visual inspection by (author ZC) resulted in 97 studies being excluded due to poor image quality, lead artifacts which impacted the LV blood pool, or failure to visualize the entire LV.

**Figure 1:**
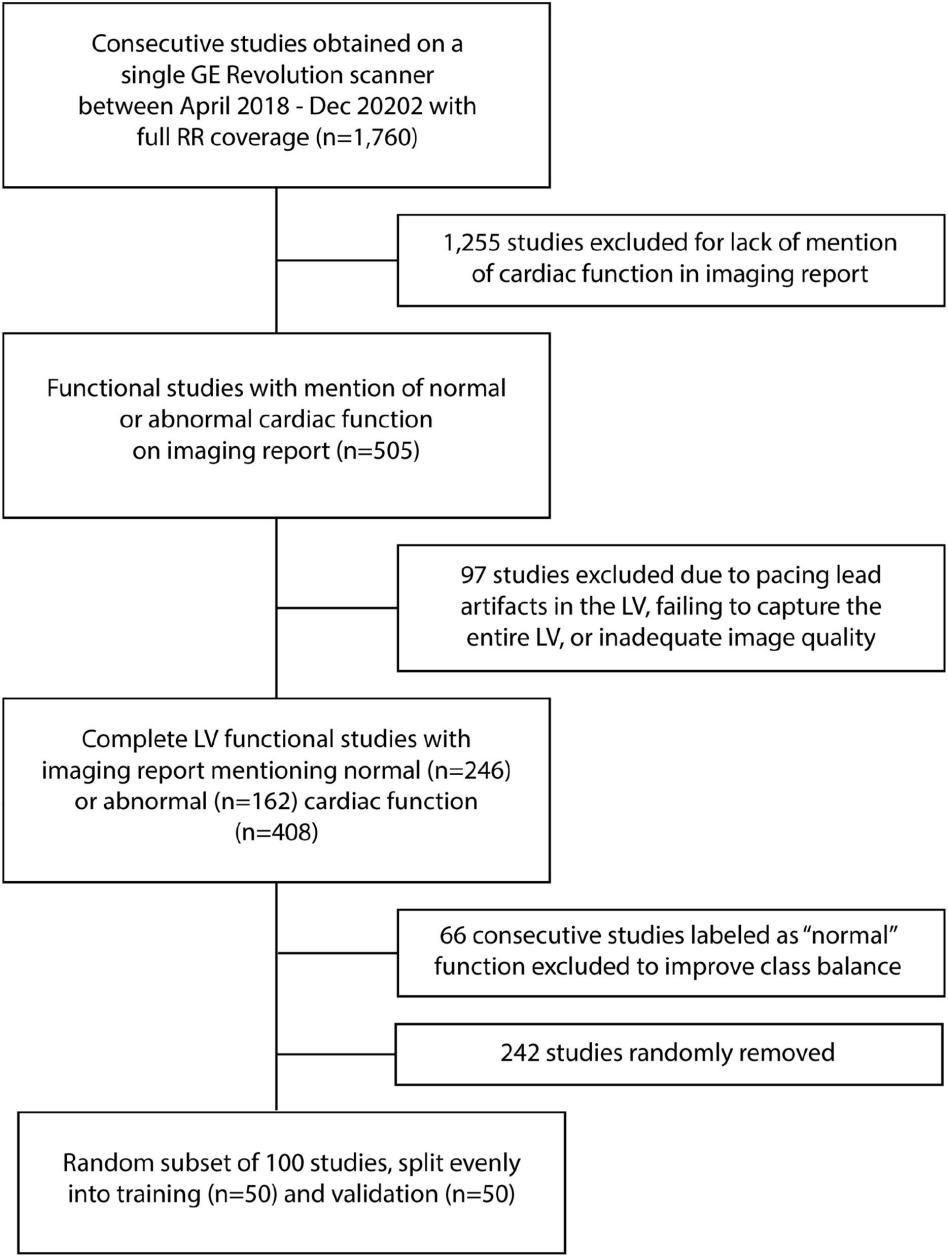
Flowchart of patient inclusion/exclusions.

Imaging reports were used to attempt to balance the study cohort. Two hundred and forty six studies were reported to have “normal” function in the report while 162 were classified as having “abnormal” function. To balance between patients with normal and abnormal function, the studies with normal function acquired at the end of the review period (acquired between August and December 2020, *n* = 66 studies total) were excluded. From the remaining *n* = 180 studies with normal function and *n* = 162 studies with abnormal function, 100 studies were randomly selected. As described below, studies selected were then visually inspected by three experts for the determination of normal/abnormal used in our study. Therefore, this step was aimed at arriving at a relatively balanced distribution of normal and abnormal studies without introducing bias into the selection process. The process is shown as a flowchart in Figure 1.

All studies had functional phases reconstructed at 10% RR intervals using the vendor default cardiac function image reconstruction method. Images were reconstructed on a 512 × 512 pixel matrix in the axial plane over a field of view of 240 ± 20 mm with 0.625 mm slice thickness.

### Expert identification of wall motion abnormalities

The CT studies were independently evaluated for WMA by three cardiovascular imagers with 15 years (A.K), 14 years (SK), and 5 years (HKN) of experience interpreting cardiac studies. For each study, wall motion at 16 AHA segment locations (not including the apical segment) was labeled, in a blinded fashion, as either (1) normal, (2) hypokinetic, (3) akinetic or dyskinetic. This was performed using movie reformats of the 4D CT dataset along standard 2D short- and long-axis views. This led to 1,600 segments being labeled. Given the limited number of hypo- and dyskinetic segments and the interobserver variability, we combined hypokinetic, akinetic and dyskinetic labels into a single “abnormal” class and only performed per-imaging plane and per-study comparison. A long-axis view was considered abnormal if it contained one or more AHA segments that were labeled abnormal. Given that three long axis videos were made per patient, this resulted in 300 long-axis videos (150 in the training and 150 in the testing cohort), each with a normal or abnormal designation. A CT study was classified as abnormal if it had one or more abnormal LAX video. For comparison to our DL-based approach, the three expert scores were combined such that a segment was labeled abnormal if there was agreement by two or more readers.

### Automated estimation of longitudinal strain along each long-axis plane

As described by Chen at al. (30), automated blood pool and long-axis views were generated by using a modified U-net architecture. Briefly, the algorithm was first trained to perform blood pool segmentation of the left atrium and ventricle. Then, an output was added after the last max-pooling layer in the downsampling path. This was used to regress the translation vector (to define the spatial position of the long-axis view) and direction vectors (to define the orientation of the view) for each of the long-axis views. The code to perform this segmentation and slice planning is available here: https://github.com/ucsd-fcrl/DL_CT_Seg-Plane_Prediction_Final_v_ZC.

**Figure 2:**
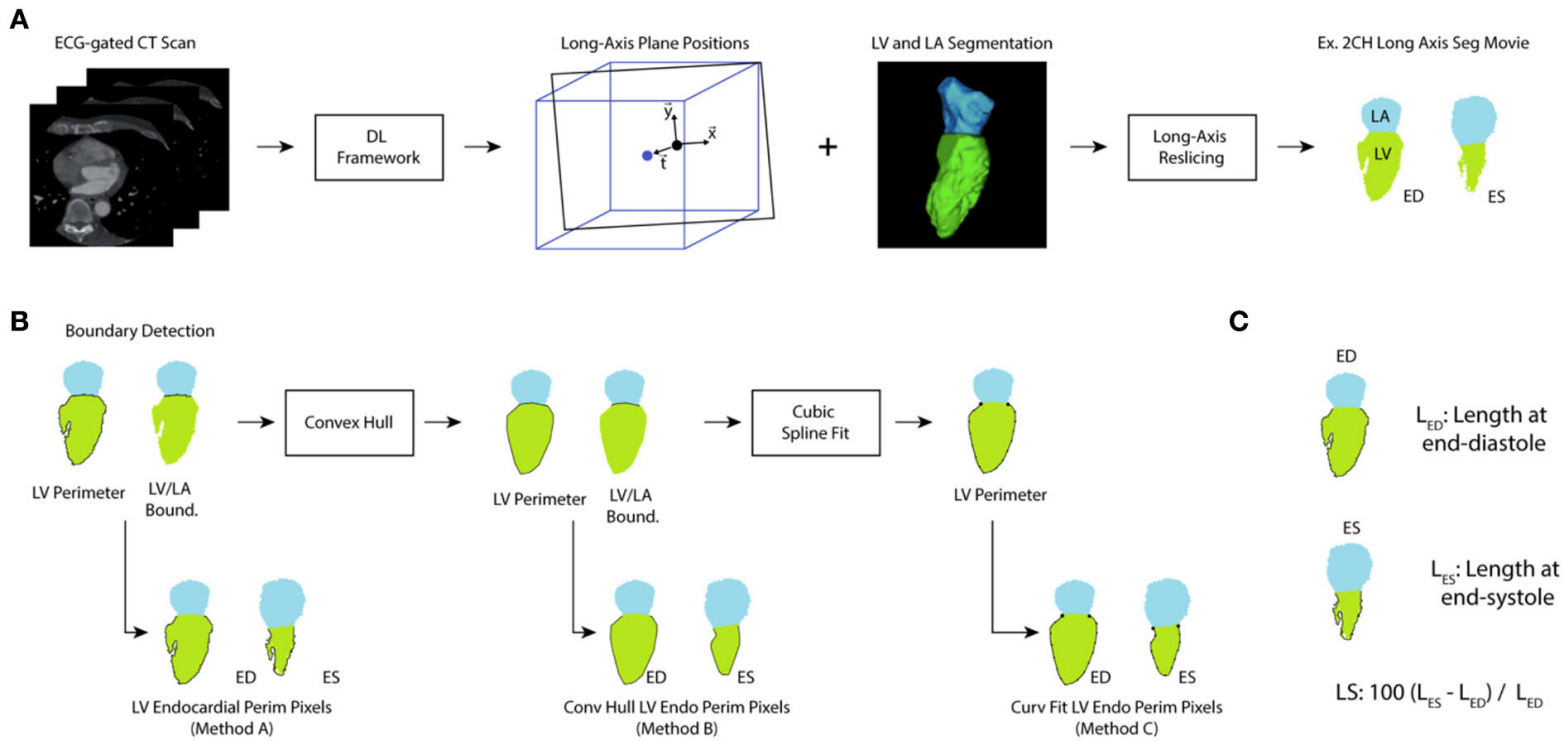
Processing of ECG-gated CT for evaluation of LS. **(A)** ECG-gated volumes are analyzed using a deep-learning (DL) framework that provides the location of the 2-chamber, 3-chamber, and 4-chamber long axis planes and delineates the LV and LA blood pools. From this information, long-axis slices of the segmentations were created throughout the cardiac cycle. **(B)** The perimeter of the left ventricle and the LV/LA boundary pixels were identified and used to extract the LV perimeter. Method A did not perform any additional processing of the perimeter. However, a convex hull was applied to correct for papillary muscle artifacts (leading to Method B). Further, a cubic splint was fit to the result of the convex hull to correct for variations in texture (Method C). **(C)** For each long-axis view and each analysis method, the length of the perimeter was measured at end-diastole (the timeframe with largest LV volume) and end-systole (the timeframe with smallest LV volume) and used to calculate LS.

The bloodpool segmentation at each of the long-axis views was evaluated and the left atrial segmentation was used to identify portions of the left ventricle bloodpool which correspond to the mitral valve. Based on this designation, the length of the LV endocardial boundary was calculated. This methodology has been previously been used with echocardiographic imaging (31, 32) and prior work in CT has measured global LS using epicardial contours (33). The process is shown in Figure 2. We expect our approach will more closely match speckle tracking echocardiography (as GLS is measured close to the endocardial boundary) rather than tagged CMR (where evaluation focuses primarily on mid-myocardial deformation) (34). Further, by measuring LS using an automated approach, our method aims to eliminate a significant source of variation (manual contouring by operators) (34).

### Papillary muscle artifacts and correction approaches

**Figure 3:**
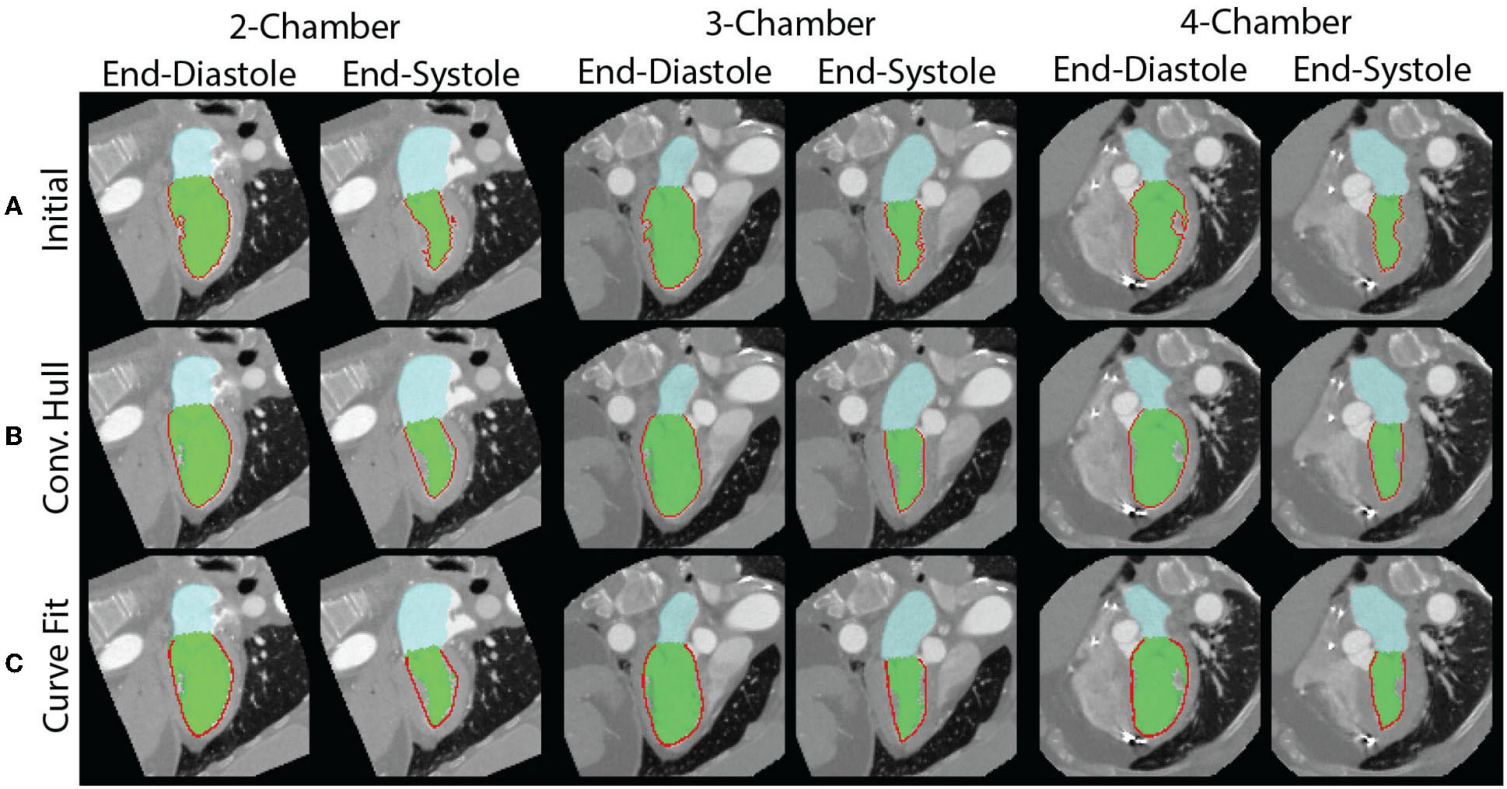
Measurement of endocardial perimeter based on the blood pool segmentation is susceptible to artifacts created by the papillary muscles. **(A)** The papillary muscles create indentations which impact the measurement of the perimeter. The end-diastolic (left) and end-systolic (right) perimeters for each of the views are shown. They are all overestimated. **(B)** By modeling the blood pool as a convex hull, we can correct for the errors from indentations created by papillary muscles. However, the perimeter measurement remains affected by the perimeter's texture. **(C)** Fitting of a curve to the perimeter avoids issues related to the surface texture.

Measuring LS directly from the segmentation was susceptible to artifacts due to the papillary muscles. An example is shown in Figure 3A. Two smoothing approaches were implemented and evaluated, First, the concave areas created by the papillary muscles were “filled in” by using the binary “close” function with a disk of 10 pixels and then fitting a convex hull to the perimeter of the endocardial bloodpool for each frame (35). An example result of this approach is shown in Figure 3.

However, there are limitations with this approach. First, the perimeter measured depends on the “texture” of the surface. This may lead to overestimation of the perimeter. Second, use of the convex hull fills the area of the papillary muscle insertion with a straight line that may underestimate the perimeter. To address these limitations, we fit a “natural” spline curve (36) to the perimeter obtained after closing and filling *via* the convex hull. Fitting was performed after downsampling the curve by a user-defined factor of 5. The result of the three methods, in the same patient as above, is shown in Figure 3. The code used to generate the different LS measures is available here: https://github.com/ucsd-fcrl/DL_CT_GLS_Final.

For all three methods, LS was calculated as the change in length over time. The unsmooth LS result as well as LS after convex hull and convex hull + curve fitting refinement were evaluated by comparing the LS estimate to the LV ejection fraction and the fractional area change (FAC) of the corresponding view.

### Determination of LS cutoffs in training cohort and evaluation in testing cohort

We varied the threshold used to determine whether a LS value (for a particular view) accurately detected the presence of a WMA, as determined by our three experts. Using the training cohort (*n* = 50), we identified the thresholds which optimized performance for each LAX view and identified the single threshold that had peak performance when applied to all LAX views. Optimal performance was based on the threshold corresponding to the upper left most point on the receiver operating characteristic (ROC) curve.

The accuracy, sensitivity, and specificity of these thresholds were then evaluated in an independent cohort of *n* = 50 patients.

### Statistical evaluation

Normally distributed values are expressed as mean ± standard deviation while non-normal values are reported using the median and interquartile range (IQR). Two-tailed categorical *z*-test was used to compare data proportions (e.g., proportions of abnormal videos) in the training and a testing cohort. To compare *R*^2^ values between fractional area change (FAC) and LS for different smoothing methods in dependent samples, the Fisher's *r*-to-*z* transformation was utilized to determine statistical significance. Statistical significance was set at *P* ≤ 0.05.

The ability of LS to detect WMA was compared against the expert labeled ground truth label and was reported *via* confusion matrix and Cohen's kappa value. Both per-long axis video and per-study comparisons were performed. Readers reviewed long-axis and short-axis movies of the cardiac cycle and labeled each AHA segment. A video was labeled as abnormal if it had one or more abnormal AHA segments present. A study was defined as abnormal if it had one or more long-axis videos labeled as abnormal. Interobserver agreement in terms of labeling wall motion as normal or abnormal between three experts was measured using Fleiss's Kappa (37) since there were more than two observers.

Anonymized long-axis images, calculated perimeters, and corresponding expert annotations will be made available upon request.

## Results

Sixty-one subjects were men and 49 were women with a mean age of 59 ± 14. Studies were obtained for evaluation of coronary disease (*n* = 50), pre-ablation assessment of pulmonary vein anatomy (*n* = 33), assessment prior to left ventricular assist device placement (*n* = 9), preoperative assessment for transcatheter aortic valve replacement (*n* = 5), and evaluation of cardiac function after chemotherapy (*n* = 3). The LV blood pool had a median intensity of 530 HU (IQR: 435–663). Out of the 1,600 segments evaluated, 27% (432/1,600) were labeled abnormal by experts. This led to 39.3% (118/300) abnormal long-axis videos and 46 studies with at least one abnormal AHA segment. There were no significant differences (all *P*-values > 0.05) between the training and testing cohorts in terms of the percentages of sex, abnormal videos, abnormal CT studies.

Median LV ejection fraction (EF) for the training and validation cohorts were 62.1 and 63.8%, respectively. In the training cohort, normal studies had an EF of 69.0% (interquartile range of 65.1–73.0%) while abnormal studies had an EF of 38.1% (IQR: 28.3–48.6%). In the validation cohort, normal studies had an EF of 67.8% (IQR: 63.6–74.2%) and abnormal studies had an EF of 49.0% (IQR: 26.0–56.0%).

Automated, DL approach successfully analyzed 48/50 patients in the training cohort and 47/50 in the testing cohort. The five failures occurred due to incorrect prediction of long-axis planes. In two of these five cases, the patients had a metal prosthetic mitral valve.

84.6% (1,354/1,600) of segments were labeled identically by all three reviewers. The interobserver agreement amongst the three observers in terms of classifying a segmental wall motion into normal vs. abnormal, measured *via* Fleiss's Kappa, was 0.746, which indicates strong agreement. Fleiss's Kappa for agreement in classifying a LAX video was 0.800 (0.791, 0.811, and 0.797 for the 2, 3, and 4 CH views, respectively) and the value for classifying a patient was 0.786.

### Correction for papillary muscle artifacts

**Figure 4:**
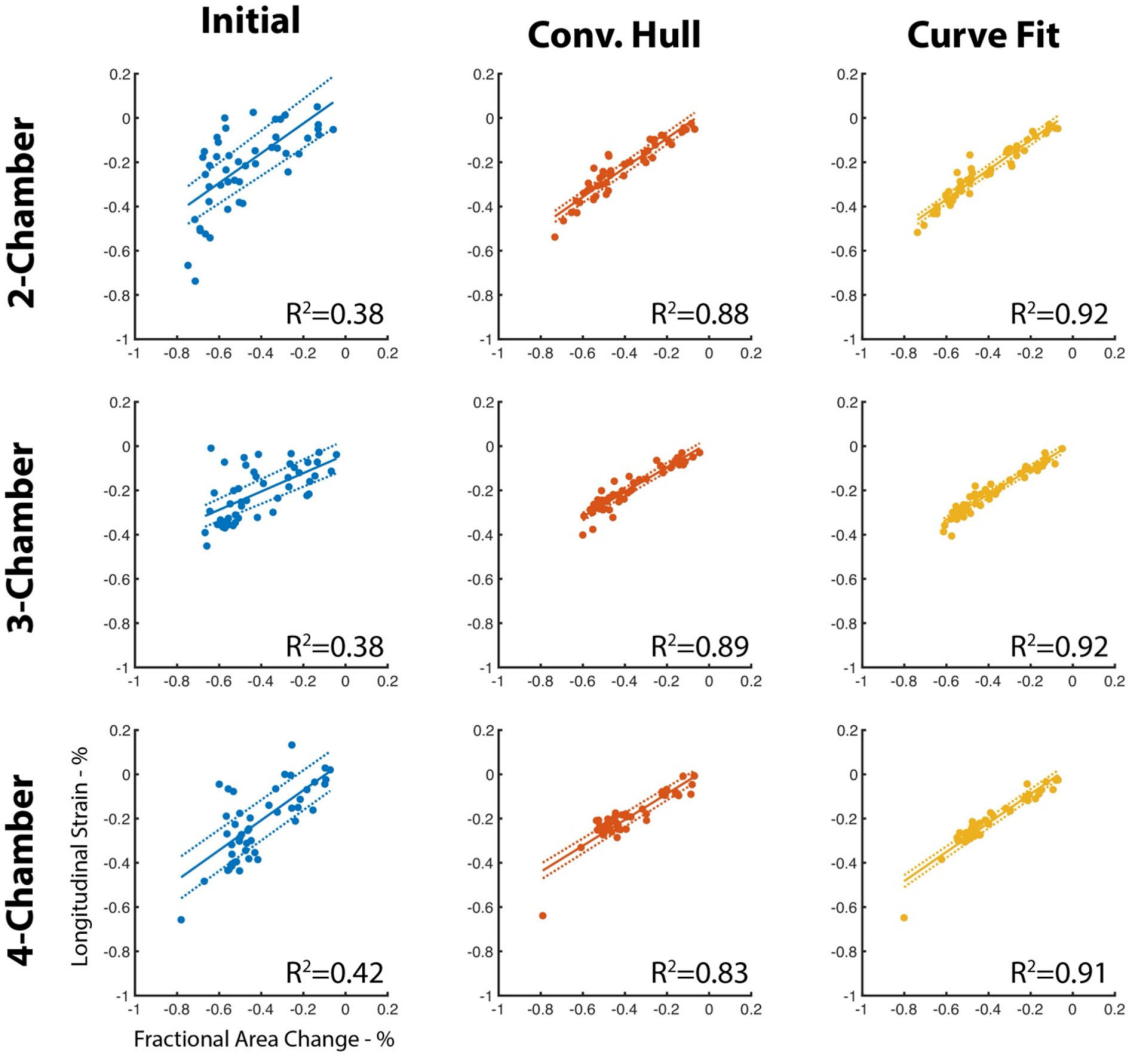
Agreement between LS and FAC increases with use of the convex hull and perimeter curve fitting. The perimeter measured using our deep learning method is susceptible to artifacts due to the insertion points of the papillary muscles and by the texture of the endocardial surface. Use of a convex hull to “fill” in the papillary insertions and curve fitting of the surface improves agreement (*R*^2^) with fractional area change of the corresponding long-axis view. Dotted lines represent the 95% confidence interval of the linear fit.

The papillary muscle artifacts and the rough endocardial surface led to poor agreement between the fractional area change and longitudinal strain (LS) when LS is measured without use of the convex hull or surface smoothing (Figure 4). Specifically, the *R*^2^ between fractional area change (FAC) and LS is between 0.38 and 0.42 depending on the long-axis view. When the convex hull is used to fill in the voids created by papillary muscles, *R*^2^ increases (0.83–0.89, Figure 4). Curve fitting of the endocardial surface leads to a further increase in *R*^2^ (0.91–0.92, Figure 4). The increase in *R*^2^ was statistically significant (*p* < 0.05) for all views.

### Determination of LS cutoffs and classification performance in training cohort

**Figure 5:**
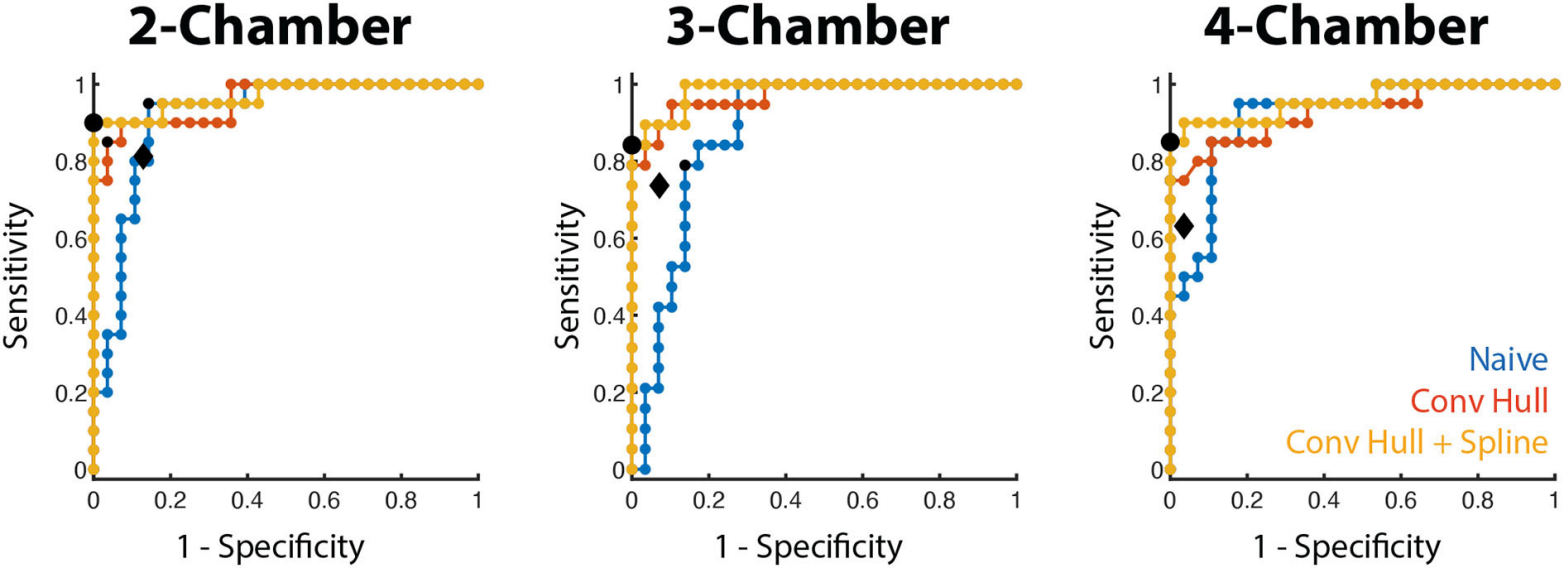
WMA classification accuracy using LS in the training cohort. Receiver operating characteristic curves for the three long-axis views are shown for the three LS methods (blue: naive, red: convex hull, orange: convex hull + curve fitting). The optimal operating point for the convex hull with curve fitting is depicted by a black dot. The operating point of the convex hull with curve fitting in the testing cohort is shown by the black diamond.

For all long-axis views, the area under the ROC curve using the convex hull and curve fitting was high (0.957–0.984, Figure 5) and the optimal threshold corresponded to a 100% specificity performance, accuracy >91.7% and sensitivity between 84.2 and 90.0% There was a small range of LS thresholds amongst LAX views with a higher cutoff identified for the 2 CH view (−0.218) relative to the 3 and 4 CH views (−0.154 and −0.166, respectively). Per-patient performance (95.8% accuracy, 90.0% sensitivity, 100% specificity) was comparable to the values obtained for each long-axis view.

We also evaluated the ability of a single threshold to classify WMA across all long-axis views. When pooled, LS thresholding had an area under the ROC of 0.965 and the use of −0.170 as the cutoff led to 92.4% accuracy, 83.0% sensitivity, and 100% specificity. This led to 95.8% accuracy, 90.0% sensitivity, and 100% specificity when classifying patients. Complete values are shown in Table 2.

**Table 2:** Use of training cohort for identification of LS cutoffs for WMA detection using the curve fitting approach.

		**Thresh**	**AUC**	**Acc**	**Sens**	**Spec**	**PPV**
Individual threshold	2 CH	−0.218	0.970 (0.914–1)	93.8 (89.9–100)	90.0 (76.9–100)	100	100
	3 CH	−0.154	0.984 (0.942–1)	91.7 (83.9–99.5)	84.2 (67.8–100)	100	100
	4 CH	−0.166	0.957 (0.892–1)	91.7 (83.9–99.5)	85.0 (69.4–100)	100	100
	Per-LAX view			92.4 (88.0–96.7)	81.4 (71.4–91.3)	100	100
	Per-patient			95.8 (90.2–100)	90.0 (76.9–100)	100	100
Single threshold	Per-LAX view	−0.170	0.965 (0.930–0.999)	92.4 (88.0–96.7)	83.1 (73.5–92.3)	100	100
	Per-patient			95.8 (90.2–100)	90.0 (76.9–100)	100	100

Thresh, optimal threshold identified for classification; AUC, area under the receiver operating characteristic curve; Sens, sensitivity; Spec, specificity; PPV, positive predictive value; 2 CH, two-chamber view; 3 CH, three-chamber view; 4 CH, four-chamber view; LAX, long-axis view. 95% confidence interval values are given in the parenthesis.

### Per-study and per-video classification performance in testing cohort

Using the convex hull and curve fitting approach, we then applied the thresholds identified in the training cohort to the testing population. The accuracy and specificity remained high (>83.0 and >87.1%, respectively) when each view was evaluated independently. Sensitivity ranged between 63.2% (4 CH view) and 81.3% (2 CH view). This led to an overall accuracy in classifying LAX views of 84.4% with a specificity of 92.0%. The use of a single threshold had similar performance (85.1% accuracy, 94.3% specificity). In both the individual and single threshold case, the per-patient accuracy was 83.0% in the testing cohort. Complete values are shown in Table 3.

**Table 3:** Performance of LS in the testing cohort using the curve-fitting approach.

		**Thresh**	**Acc**	**Sens**	**Spec**	**PPV**
Individual threshold	2 CH	−0.218	85.1 (74.9–95.3)	81.3 (62.1–100)	87.1 (75.3–98.9)	76.5 (56.3–96.6)
	3 CH	−0.154	85.1 (74.9–95.3)	73.7 (53.5–93.5)	92.9 (83.3–100)	87.5 (71.3–100)
	4 CH	−0.166	83.0 (72.2–93.7)	63.2 (41.5–84.9)	96.4 (89.6–100)	92.3 (77.8–100_
	Per-LAX view		84.4 (78.4–90.4)	72.2 (60.3–84.2)	92.0 (86.2–97.7)	84.8 (74.4–95.2)
	Per-patient		83.0 (72.2–93.7)	84.2 (67.8–100)	82.1 (68.0–96.3)	76.2 (58.0–94.4)
Single threshold	Per-LAX view	−0.170	85.1 (79.2–91.0)	70.4 (58.2–82.6)	94.3 (89.4–99.1)	88.4 (78.8–98.0)
	Per-patient		83.0 (72.2–93.7)	79.0 (60.6–97.3)	85.7 (72.8–98.7)	79.0 (60.6–97.3)

Thresh, optimal threshold identified for classification; AUC, area under the receiver operating characteristic curve; Sens, sensitivity; Spec, specificity; PPV, positive predictive value; 2 CH, two-chamber view; 3 CH, three-chamber view; 4 CH, four-chamber view; LAX, long-axis view. 95% confidence interval values are given in the parenthesis.

## Discussion

We demonstrate how deep learning (DL) segmentation of the left atrial and left ventricular bloodpools can be combined with automated prediction of the long-axis imaging planes to automatically calculate longitudinal strain along each long-axis view and detect wall motion abnormalities. In this study, we applied the previously trained DL tool to our CT studies without retraining or refinement and developed steps to extract LS from the resulting data. To the best of our knowledge, this is the first study to automatically quantify LS along long-axis views from ECG-gated cardiac CT angiograms. To demonstrate the clinical utility, we evaluated the ability of automated LS to detect WMA. When applied to the testing cohort, the LS identified WMA with accuracy > 83.0% and specificity > 92.9%.

A single LS threshold value of −17.0% had similar performance during the training phase as unique thresholds for each long-axis view and higher performance in the testing cohort. This LS cutoff is similar to those previously reported in other populations and with other imaging methods. In a meta-analysis of chemotherapy-induced cardiotoxicity, Oikonomou et al. reviewed studies which had high-risk cutoff values of −21.0 to −13.8% (3). Similarly, Kearney et al. found LS in controls to be −21 ± 2% while patients with AS had LS between −18 and −15% depending on the AS severity (4) and Zhu et al. found mortality in AS patients was higher in those with LS > −15.2% (5). Recently, Chen et al. reported another automated method to detect wall motion abnormalities using ECG-gated CT which relies on a volume rendering approach (38). Our results are slightly lower than the per-patient accuracy (93.5%), sensitivity (91.9%), and specificity (94.7%) reported in this prior work. This is likely due to the fact that LS provides a single metric of performance which may mask subtle abnormalities.

This method could add to the clinical interpretation of cardiac CT angiograms by serving as an aid for expert readers. It is also likely that providing the LS score for each view is of value. For example, reporting the LS score along with the relevant cutoff would enable the expert to gain a sense of both the prediction of the algorithm as well as the confidence of the prediction. Also, it is possible that a high sensitivity threshold provides more clinically useful predictions, especially if applied to patients in a screening type of setting. However, this utility is left for future studies. Full R-R ECG-gated imaging has higher dose than obtaining only a single phase. This can be partially mediated by dose modulation. Twenty-five percentage of the studies evaluated in this study had mA reduction of >50% during the cardiac cycle without an impact on clinical interpretability.

While the development of the deep learning segmentation required specialized graphics hardware, the use of the DL and the subsequent LS processing can be easily incorporated into a clinical pipeline and can be readily performed on conventional computers. Further, there are additional metrics that can be readily obtained from this tool, such as the mitral annular plane systolic excursion (MAPSE). However, the extraction and utility of such metrics is left for future studies.

As mentioned, 3D methods to measure endocardial displacement using ECG-gated CT have been previously described (18–21). Solving for endocardial displacement is computationally intensive and delineating the endocardial surface throughout the chamber can be time-intensive. However, recent work aims to avoid these limitations (39). Therefore, our streamlined, automated approach could serve as an initial check to determine whether more extensive assessment is needed.

Our study had several limitations. First, our single site/scanner study only evaluated studies which had global function reported on radiology reports. These factors could introduce biases and motivate a dedicated study to validate our findings in an external, broader cohort across multiple vendors. However, detailed evaluation of wall motion abnormalities in a standardized, AHA segment fashion is not readily available. Second, the DL segmentation failed to produce accurate segmentations and/or long-axis imaging planes in 5/100 patients (*n* = 2 in the training cohort and *n* = 3 in the testing cohort). The 95% success rate is likely sufficient for clinical use, especially given that the result of the DL blood pool segmentation and long-axis planes can be displayed to the reader for review. Our study excluded studies with low image quality, lead artifacts, and incomplete coverage of the LV as the DL method developed by Chen et al. relied on these exclusion criteria (25). Therefore, future work is needed to determine the failure rate in a larger, more diverse, dataset. Further, our approach identifies WMA using LS since the DL segmentation only provides endocardial boundary information. If epicardial segmentations were available, then other metrics such as regional wall thickening could be measured. As a retrospective study, paired echocardiography and MRI data were not available. Future work should directly compare LS measured with CT to these more-conventional methods. Lastly, LS is correlated with other metrics of function such as fractional area change (FAC) and ejection fraction (EF). Our study was not designed nor powered to identify whether LS is a better independent predictor of WMA than these other metrics but others have documented the utility of LS (7, 9).

In conclusion, longitudinal strain (LS), typically measured with MRI or echocardiography, has been previously shown to be diagnostic and prognostic of several patient populations. We leverage a recently developed deep learning approach to automate LS estimation in ECG-gated CT angiograms (cineCT) and demonstrate that LS can be used to detect wall motion abnormalities.

## Data availability statement

The raw data supporting the conclusions of this article will be made available by the authors, without undue reservation.

## Ethics statement

The studies involving human participants were reviewed and approved by UCSD Institutional Review Board. Written informed consent for participation was not required for this study in accordance with the national legislation and the institutional requirements.

## Author contributions

HL, ZC, and FC performed the data analysis while AK, SK, and HN performed the visual evaluation of the data. All authors contributed to drafting the revising of the manuscript.
